# Genomic insights into the conservation status of the Idle Crayfish *Austropotamobius bihariensis* Pârvulescu, 2019: low genetic diversity in the endemic crayfish species of the Apuseni Mountains

**DOI:** 10.1186/s12862-024-02268-5

**Published:** 2024-06-11

**Authors:** Lena Bonassin, Lucian Pârvulescu, Ljudevit Luka Boštjančić, Caterina Francesconi, Judith Paetsch, Christelle Rutz, Odile Lecompte, Kathrin Theissinger

**Affiliations:** 1 Department of Computer Science, Centre de Recherche en Biomédecine de Strasbourg, UMR 7357, University of Strasbourg, CNRS, Rue Eugène Boeckel 1, 67000 ICube, Strasbourg, France; 2 Present Address: LOEWE Centre for Translational Biodiversity Genomics (LOEWE-TBG), Senckenberganlage 25, 60325 Frankfurt am Main, Germany; 3 Institute for Environmental Sciences, Department of Molecular Ecology, Rhineland-Palatinate Technical University Kaiserslautern Landau, Fortstr. 7, 76829 Landau, Germany; 4 Department of Biology-Chemistry, Faculty of Chemistry, Biology, Geography, West University of Timisoara, Str. Pestalozzi 16A, 300115 Timisoara, Romania; 5 Crayfish Research Centre, Institute for Advanced Environmental Research, West University of Timisoara, Oituz 4, 300086 Timisoara, Romania; 6 Department of Biogeography, University of Trier, Behringstraße 21, D-54296 Geozentrum, Trier, Germany; 7 Institute for Insect Biotechnology, Justus Liebig University Giessen, Heinrich-Buff-Ring 26, D-35392 Giessen, Germany

**Keywords:** ddRAD-seq, Endemic species, Idle Crayfish, Genetic diversity, Apuseni mountains

## Abstract

**Background:**

Biodiversity in freshwater ecosystems is declining due to an increased anthropogenic footprint. Freshwater crayfish are keystone species in freshwater ecosystems and play a crucial role in shaping the structure and function of their habitats. The Idle Crayfish *Austropotamobius bihariensis* is a native European species with a narrow distribution range, endemic to the Apuseni Mountains (Romania). Although its area is small, the populations are anthropogenically fragmented. In this context, the assessment of its conservation status is timely.

**Results:**

Using a reduced representation sequencing approach, we identified 4875 genomic SNPs from individuals belonging to 13 populations across the species distribution range. Subsequent population genomic analyses highlighted low heterozygosity levels, low number of private alleles and small effective population size. Our structuring analyses revealed that the genomic similarity of the populations is conserved within the river basins.

**Conclusion:**

Genomic SNPs represented excellent tools to gain insights into intraspecific genomic diversity and population structure of the Idle Crayfish. Our study highlighted that the analysed populations are at risk due to their limited genetic diversity, which makes them extremely vulnerable to environmental alterations. Thus, our results emphasize the need for conservation measures and can be used as a baseline to establish species management programs.

**Supplementary Information:**

The online version contains supplementary material available at 10.1186/s12862-024-02268-5.

## Background

Freshwaters are one of the most diverse ecosystems on the planet with exceptionally high levels of endemism [1]. Unfortunately, freshwater biodiversity is declining rapidly, faster than that of terrestrial or marine, with small endemic populations with limited distribution being especially affected [2]. Among the other taxa, freshwater crayfish are keystone species with a fundamental role in determining the structure and function in freshwater ecosystems [3]. Moreover, crayfish have a high cultural significance, especially in Europe [4]. However, many native crayfish populations are in decline and nearing extinction [5]. Native crayfish species richness in Europe is relatively low, with only six native species present [6]. Nevertheless, the species’ genetic diversity is high, especially within the genus *Austropotamobius* [7–9]. The Idle Crayfish, *Austropotamobius bihariensis*, Pârvulescu, 2019, is an endemic freshwater crayfish species with the smallest distribution range restricted to the Apuseni Mountains in Romania [10]. The small distribution range covers tributaries of the three Criș rivers in the Apuseni Mountains characterised by habitats with clean waters in the mountainous and sub-mountainous regions [10]. Being a recently described species, the conservation status of *A. bihariensis* is not yet finally determined [11]. With the other *Austropotamobius* species, it is the most vulnerable among native European freshwater crayfish species, being threatened by water quality deterioration, urbanisation, and other anthropogenic influences [12, 13]. Compared to other native species, the genus *Austropotamobius* has lower dispersal capacity, lower reproductive output and higher oxygen demand [14, 15]. Moreover, the invasive spiny-cheek crayfish *Faxonius limosus* (Rafinesque, 1817) is spreading through the Romanian rivers, carrying the crayfish plague pathogen *Aphanomyces astaci* [16–19]. This pathogen has already caused the devastation of several native crayfish populations throughout Europe, and its presence has been recently confirmed among *A. bihariensis* populations [20, 21].

Species with restricted distribution and limited dispersal capabilities are threatened by extinction due to loss of habitat, genetic variation, and invasive species [22]. Endemic species present in a narrow geographical range are particularly vulnerable and are often characterised by a small population size [23]. In small populations, genetic diversity is quickly declining due to genetic forces, posing a threat to the long-term survival of the species [24]. Specifically, genetic variability is important for preserving the adaptive potential of a species, its reproductive success and disease resistance, especially in response to environmental changes [25, 26]. Genetic characterisation of individuals within and amongst populations allows the identification of genetic population structure and gene flow for defining genetically similar conservation units [27]. This information can then be used to conduct informed management actions such as habitat restoration or species translocation [28].

The assessment of genetic diversity is an initial step towards conservation actions. Genomic data allows to characterise and monitor genetic diversity, maximising the information obtained from each individual [29]. Previous studies on *A. bihariensis* and other European freshwater crayfish taxa focused on the phylogenetic analysis of mitochondrial DNA markers [9], and the population diversity was assessed based on a small number of microsatellite loci [12]. A small number of loci can limit the characterisation of the genetic diversity of species. This limitation can be overcome by using genome-wide assessments [29]. When a reference genome is unavailable, a reduced representation DNA sequencing (RRS), which sequences a random fraction across the entire genome, is a suitable approach to provide key insights into the genomic structure of a population. In particular, ddRADseq (double digest DNA restriction-site-associated DNA sequencing) provides a large single nucleotide polymorphism (SNP) dataset from a subset of the genome [28]. Unlike microsatellites, SNPs are more abundant and uniformly distributed across the genome, being found in both non-coding and coding regions of the genome, and thus increase the sensitivity of the analysis and robustness of population genetic estimates compared to microsatellite markers. Therefore, SNPs are appropriate markers for the assessment of demographic as well as functional processes [28].

Here, we conducted the first population genomic analyses of the endemic freshwater crayfish species *A. bihariensis* using a large SNP dataset to aid the assessment of the species conservation status. We performed ddRAD sequencing to obtain an insight into the genomic variants and genetic diversity present in 13 populations, belonging to five river basins across the entire distribution range of this endemic species. Due to the low dispersal capability of the species, we hypothesised that the population structure is reflected by the river basins. Based on the identified SNPs, we also aimed to uncover unique genomic variants to identify populations of the highest conservation priority.

## Methods

### Sample collection and DNA extraction

**Fig. 1:**
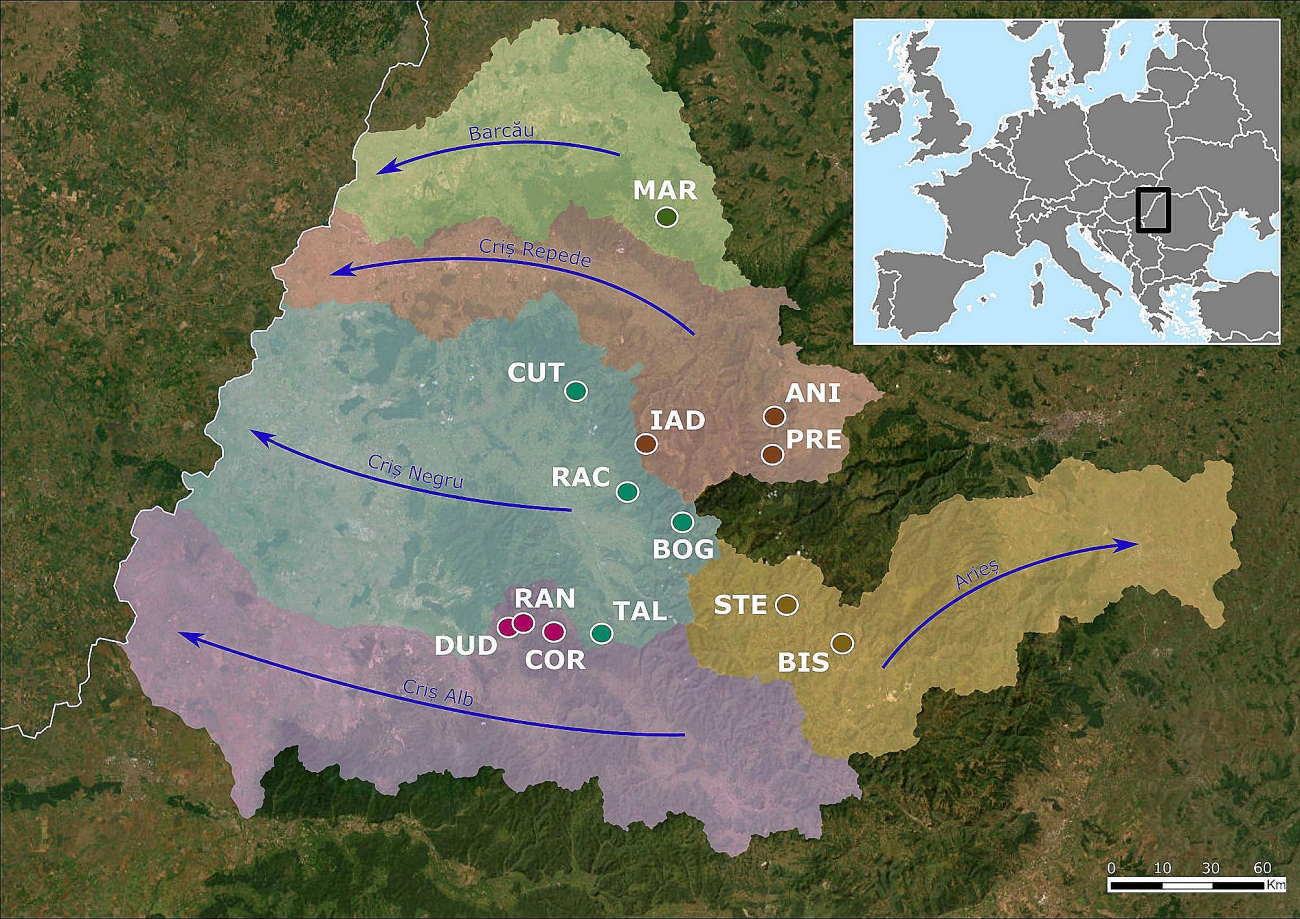
Distribution map of the sampled locations. Colours denote different river basins and arrows the direction of river flow. Population acronyms: DUD – Dudușoaia, COR – Corbului, TAL – Tâlniciorii, BOG – Boga, RAC – Racu, CUT – Cuților, IAD – Iadei, PRE – Preluca, ANI – Anișelului, MAR – Mare, STE - Starpă, BIS – Bistrii. The base map layout is provided by Earthstar Geographics (https://www.terracolor.net) and river basin boundaries were delineated using the HydroBASINS database (https://www.hydrosheds.org)

Tissue samples from 235 individuals were obtained by collecting one pereopod from each individual and tissue was stored in 96% ethanol at 4 °C until DNA extraction. Sample collection was conducted at 13 locations belonging to five river basins (Table S1) across the species distribution range (Fig. 1), obtaining between 10 and 20 individuals per population (Table S1). Considering the high number of individuals needed for population genetic studies, the sampling was not conducted in populations known to have a low number of individuals. DNA was extracted using the salting out protocol [30] with the following modifications: the digestion of the tissue was performed for 3 h at 65 °C and 400 rpm, to remove the proteins and cellular debris the samples were centrifuged at 5000 g for 10 min, and to precipitate the DNA the samples were centrifuged at 5000 g for 5 min. Finally, the DNA pellet was resuspended in 60 µL nuclease-free water. DNA was quantified using the QuantiFluor® dsDNA System on the Quantus™ Fluorometer (Promega, USA).

### ddRAD sequencing

ddRAD libraries were produced by IGA Technology Services (Udine, Italy) using a custom protocol with minor modifications with respect to Peterson’s double digest restriction-site associated DNA preparation [31]. The enzyme pair was selected based on *in silico* analysis of 24 Gb of PacBio HiFi reads of the species (unpublished data). Genomic DNA was fluorometrically quantified using Qubit 2.0 Fluorometer (Invitrogen, Carlsbad, CA, USA) and normalised to a uniform amount. It was then double digested with 2.4 U of both PstI and EcoRI endonucleases (New England BioLabs, USA) in 30 µL reaction supplemented with CutSmart Buffer and incubated at 37 °C for 90 min, then at 75 °C for 20 min. Fragmented DNA was subsequently ligated with 180 U of T4 DNA ligase (New England BioLabs, USA) and 2.5 pmol of overhang barcoded adapters for both cut sites in a 50 µL reaction incubated at 23 °C for 60 min and at 20 °C for 60 min, followed by 20 min at 65 °C. Samples were pooled on multiplexing batches and purified with 1.5 volumes of AMPureXP beads (Agencourt). For each pool, targeted fragment distributions were collected using BluePippin (Sage Science Inc., USA) with a set range 400 –550 bp. The gel eluted fraction was amplified with indexed primers using Phusion High-Fidelity PCR Master Mix (New England BioLabs, USA) in a final volume of 50 µL and subjected to the following thermal protocol: [95 °C, 3 min] − [95 °C, 30 s, -60 °C, 30 s, -72 °C, 45 s] x 10 cycles − [72 °C, 2 min]. PCR products were purified with 1 volume of AMPureXP beads (Agencourt). The resulting libraries were checked with both Qubit 2.0 Fluorometer (Invitrogen, Carlsbad, CA) and Bioanalyzer DNA assay (Agilent technologies, Santa Clara, CA). Libraries were sequenced with 150 cycles in paired-end mode on a NovaSeq 6000 instrument following the manufacturer’s instructions (Illumina, San Diego, CA).

### ddRADseq data processing

Demultiplexing of raw Illumina reads was performed using the process_radtags utility included in Stacks v2.61 [32]. Sequence quality was assessed using FastQC v0.11.9 [33] and MultiQC v1.9 [34]. *De novo* assembly was performed in Stacks v2.62 and the parameters for the assembly were selected following the recommendations by Paris et al. [35]. For the *de novo* building of loci, creation of a catalog of loci and SNP calling, the pipeline module denovo_map.pl included in Stacks v2.62 was used with the following parameters: -m 6, -M 2 and -n 2. Using Plink v1.90 [36], SNPs and individuals with a missing call frequency greater than 0.1, and SNPs with minor allele frequency lower than 0.05 were filtered out. The filtered SNPs and individuals were used to create a whitelist and run the populations module in Stacks v2.62.

### Population genetic diversity

The population genetic diversity was assessed by calculating the percentage of polymorphic loci (P), number of private alleles, observed heterozygosity (H_O_), expected heterozygosity (H_E_), and inbreeding coefficient (F_IS_) based on SNPs in the populations module in Stacks v2.62. The analysis was first done by assigning individuals to populations and then by assigning individuals to river basins. The effective population size (N_e_) was estimated using NeEstimator v2 [37]. To obtain more reliable results, N_e_ was calculated using the LD method and heterozygote excess method, and 95% Cis were calculated by a jackknife-on-samples method. To avoid bias caused by rare allele presence, N_e_ was calculated at a Minor Allele Frequency (MAF) equal or smaller than 0.02 and 0.01.

### Population genetic structure

The population differentiation was estimated by pairwise comparisons of the fixation coefficient (F_ST_) calculated in the populations module in Stacks v2.62. The genetic structure was assessed with principal component analysis (PCA) and Bayesian clustering algorithm implemented in fastStructure [38]. PCA was performed using Plink v1.90 and plotted using the ggplot2 R package [39]. We performed the fastStructure analysis for K values between 1 and 12 to determine the most likely value for K determined by marginal likelihood. The results were visualised using the pophelper R package [40]. FineRADstructure [41] was used to observe co-ancestry among individuals and populations based on haplotypes with default parameters. Final editing of the resulting graphics was done in Inkscape 1.2.1 [42].

### Ethical statement

All tissue samples involved in this study were taken in accordance with international ethical guidelines. No animal was killed, and after collecting the sample, the animal was released exactly where it was caught. Also, the necessary approvals were requested and obtained, according to the legislation in force in the area: Romanian Academy (1/CJ/13.01.2021), Romanian Ministry of Water and Forests (DGB/2/R5787/16.08.2022), Apuseni Nature Park Administration (199/09.09.2022), National Agency for Protected Areas (882/15.09.2022), Environmental Protection Agencies in the geographical area (8027/26.07.2022, 76/20.09.2022, 53/20.09.2022, 29/27.10.2022, 77/28.10.2022).

## Results

### ddRAD data assembly

We sequenced ddRAD libraries from 235 individuals across 13 populations (Fig. 1, Table S1). In total 3 371 654 698 reads were obtained with a length of 135 bp. After quality filtering of the reads, in total 3 265 444 937 reads were retained, ranging from 872 095 to 92 642 412 reads per individual, with a mean of 13 895 510 reads (Table S2). In total, 2 042 818 loci were assembled with a mean length of 260.78 bp and 1 381 639 SNPs were identified. After SNP and individual missingness filtering, RAN population was removed due to too much missing data. The final dataset consisted of 4 875 SNPs and 205 individuals from 12 populations (Fig. 1; Table 1).

### Population genetic diversity

The observed overall genetic diversity of *A. bihariensis* populations was similar on a population level (Table 1) and at the river basin level (Table 2). Considering all populations, the percentage of polymorphic loci ranged between 0.212% and 0.464%. On the population level, only DUD had private alleles (*n* = 12). On the river basin level, private alleles were identified in the Alb (*n* = 77) and Negru (*n* = 10) river basins. The values for observed heterozygosity (H_O_) ranged from 0.164 to 0.311, with the lowest value for the ANI population and the highest for TAL. The H_O_ values for river basins ranged from 0.178 (Criș Repede basin) to 0.278 (Arieș basin). The values of expected heterozygosity (H_E_) ranged from 0.149 (ANI) to 0.294 (RAC). H_E_ values for river basins ranged from 0.177 (Criș Repede basin) to 0.313 (Criș Alb basin). The inbreeding coefficient (F_IS_) ranged from -0.058 to 0.011 for the populations and from -0.058 to 0.109 for the river basins, respectively.

**Table 1:** River basins and populations used in genetic analyses, number of individuals per population, percentage of polymorphic loci, number of private alleles, observed (H_O_) and expected (H_E_) heterozygosity and inbreeding coefficient (F_IS_)

River basin	Population	Acronym	*N*	Polymorphic loci %	Private alleles	H_O_	H_E_	F_IS_
Criș Alb	Dudușoaia	DUD	20	0.409	12	0.273	0.259	-0.001
Criș Alb	Corbului	COR	10	0.435	0	0.313	0.292	0.002
Criș Negru	Racu	RAC	17	0.459	0	0.321	0.294	-0.031
Criș Negru	Tâlniciorii	TAL	18	0.464	0	0.331	0.290	-0.058
Criș Negru	Cuților	CUT	19	0.303	0	0.210	0.198	-0.011
Criș Negru	Boga	BOG	20	0.342	0	0.238	0.232	0.011
Criș Repede	Iadei	IAD	18	0.266	0	0.181	0.173	-0.002
Criș Repede	Preluca	PRE	19	0.317	0	0.179	0.172	-0.001
Criș Repede	Anișelului	ANI	7	0.212	0	0.164	0.149	0.007
Barcău	Mare	MAR	17	0.383	0	0.251	0.217	-0.058
Arieș	Bistrii	BIS	20	0.381	0	0.210	0.190	-0.022
Arieș	Starpă	STE	20	0.309	0	0.203	0.190	-0.007

**Table 2:** Number of individuals per river basin, percentage of polymorphic loci, number of private alleles, observed (H_O_) and expected (H_E_) heterozygosity and inbreeding coefficient (F_IS_) of individuals grouped by river basins

River basin	*N*	Polymorphic loci %	Private alleles	H_O_	H_E_	F_IS_
Criș Alb	30	0.475	77	0.287	0.313	0.096
Criș Negru	72	0.498	10	0.272	0.307	0.109
Criș Repede	42	0.339	0	0.178	0.177	0.010
Barcău	17	0.383	0	0.252	0.217	-0.058
Arieș	40	0.408	0	0.207	0.205	0.017

The results of population size estimation are shown in Table 3. Across the majority of the investigated populations, the effective population size estimated for 0.02 and 0.01 MAF with LD method ranged from 3.1 to 86.3, while estimated with heterozygote excess method ranged from 6.9 to 1033.3. The estimates were indefinable (∞) for the populations BOG, ANI and COR estimated with both methods. For the majority of the populations, 95% CIs were wide, with the upper limit indefinable (∞).

**Table 3:** Effective population size estimation based on linkage diseqilibrium (N_e_LD) and heterozygote excess (N_e_b) for 0.02 and 0.01 minor allele frequency (MAF) and 95% CI based on jackknifing method – N_e_ estimator. Population acronyms: DUD – Dudușoaia, COR – Corbului, TAL – Tâlniciorii, BOG – Boga, RAC – Racu, CUT – Cuților, IAD – Iadei, PRE – Preluca, ANI – Anișelului, MAR – Mare, STE - Starpă, BIS – Bistrii

Population	*N*_e_LD	95% CI	*N*_e_b	95% CI
DUD	21.0	6.8–∞	1033.3	48.0–∞
COR	∞	23.4–∞	∞	56.8–∞
BIS	3.1	1.0–78.8	17.3	13.0–25.9
STE	72.6	18.7–∞	47.4	22.2–∞
MAR	20.1	10.7–21.1	6.9	6.2–8
RAC	86.3	27.3–88.1	14.9	11.9–20.1
TAL	26.2	8.3–99.2	8.3	7.3–9.6
CUT	55.9	21.8–∞	28.9	17.0–102.5
BOG	∞	∞–∞	∞	∞–∞
IAD	39.2	10.8–∞	139.9	28.4–∞
PRE	9.6	3.3–22.4	449.4	39.3–∞
ANI	∞	∞–∞	∞	336.9–∞

### Population genetic structure

**Fig. 2:**
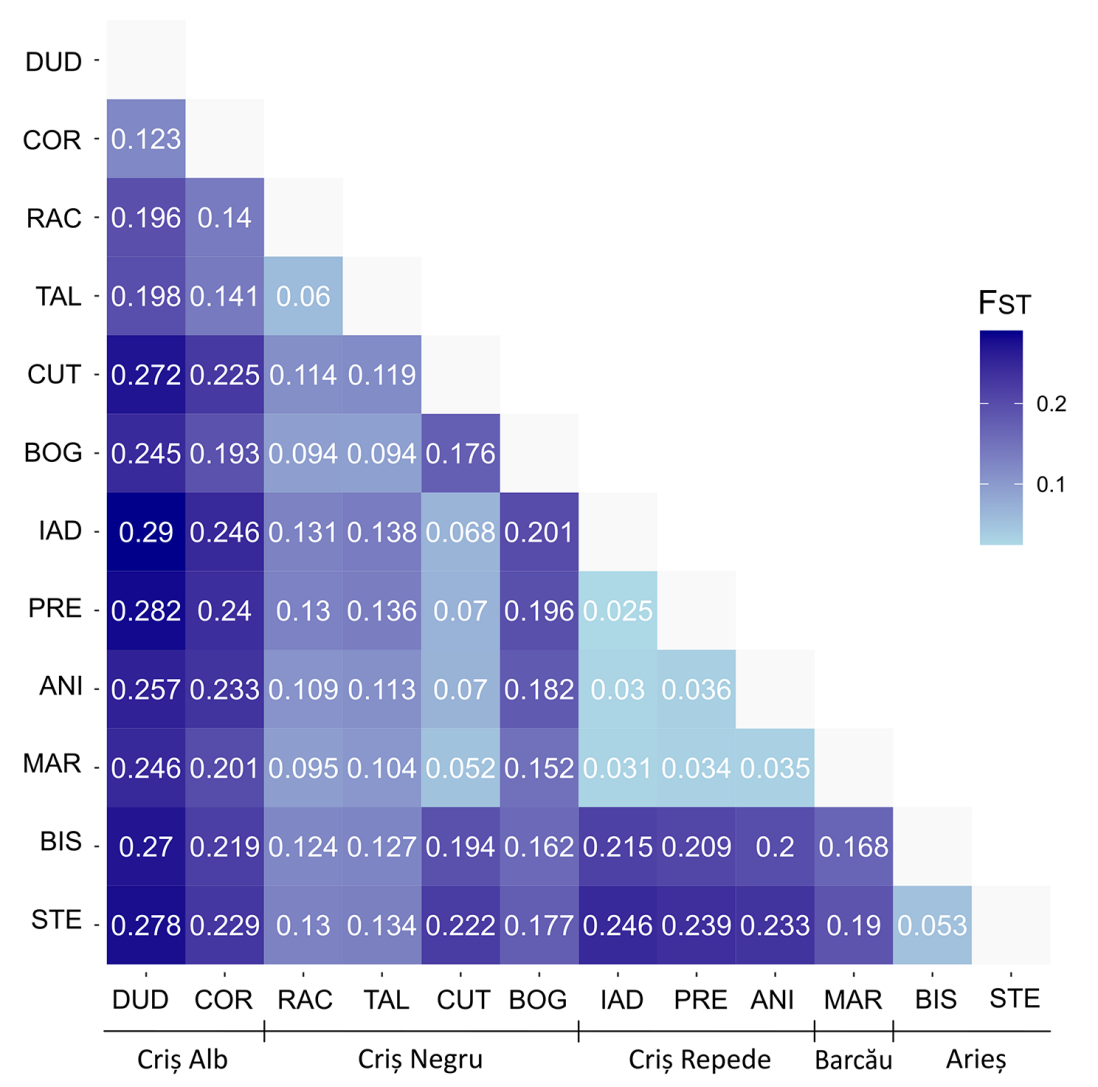
Fixation coefficient (F_ST_) between each population pair. Darker blue indicates higher F_ST_ values. Population acronyms: DUD – Dudușoaia, COR – Corbului, TAL – Tâlniciorii, BOG – Boga, RAC – Racu, CUT – Cuților, IAD – Iadei, PRE – Preluca, ANI – Anișelului, MAR – Mare, STE – Starpă, BIS – Bistrii

**Fig. 3:**
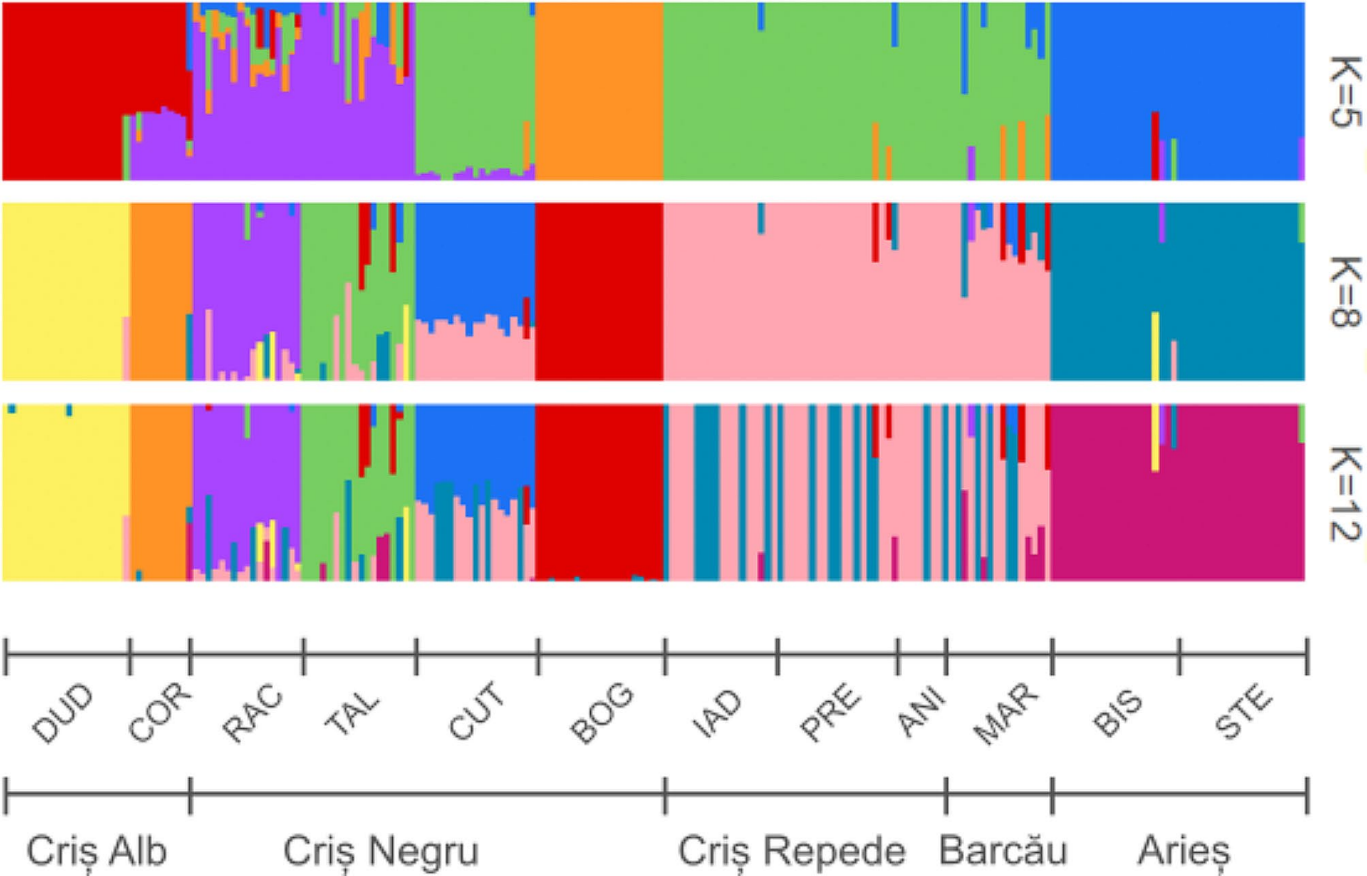
Population structure based on fastStructure analysis for K = 5, 8, and 12. Different colours represent different genetic clusters. Each column represents one individual. Population acronyms: DUD – Dudușoaia, COR – Corbului, TAL – Tâlniciorii, BOG – Boga, RAC – Racu, CUT – Cuților, IAD – Iadei, PRE – Preluca, ANI – Anișelului, MAR – Mare, STE – Starpă, BIS – Bistrii. Columns with different colours indicate admixture of populations

The pairwise fixation index (F_ST_) ranged from 0.025 (PRE – IAD) to 0.29 (DUD – IAD) (Fig. 2). The highest F_ST_ is present in the DUD population, while the lowest differentiation is seen in the MAR population. The structuring of population with K = 5 (number of river basins), K = 8 (highest marginal likelihood revealed by fastStructure analysis) and K = 12 (number of populations) is shown in Fig. 3. With K = 5, the DUD and COR populations shared one cluster, in accordance with their belonging to the Criș Alb river basin. A second distinct cluster was formed by the populations of the Arieș river basin. RAC and TAL populations, originating from Criș Negru river basin, form another distinct cluster with the largest number of admixed individuals. All individuals from BOG population belonged to one distinct cluster. CUT, MAR, IAD, PRE and ANI populations belonged to one other cluster combining populations from river basins Criș Negru (CUT), Barcău (MAR) and Criș Repede (IAD, PRE, ANI) (Fig. 3). With K = 8, the individuals from DUD, COR populations (Criș Alb basin), and BOG, RAC and TAL (Criș Negru basin) formed each their own unique cluster (Fig. 3). Some individuals from the RAC and TAL populations showed genetic admixture and belonging to multiple clusters. The BIS and STE populations together formed one cluster in accordance with their geographical location, both belonging to the Arieș river basin. The MAR population (Barcău basin), and ANI, PRE, IAD populations from Criș Repede basin formed one cluster, shared with all the individuals from the CUT population (Criș Negru basin). With K = 12, the clustering of the populations was the same as for K = 8, except for some individuals from populations CUT (Criș Negru basin), MAR (Barcău basin), IAD, PRE, ANI (Criș Repede basin) which split into a separate cluster.

**Fig. 4:**
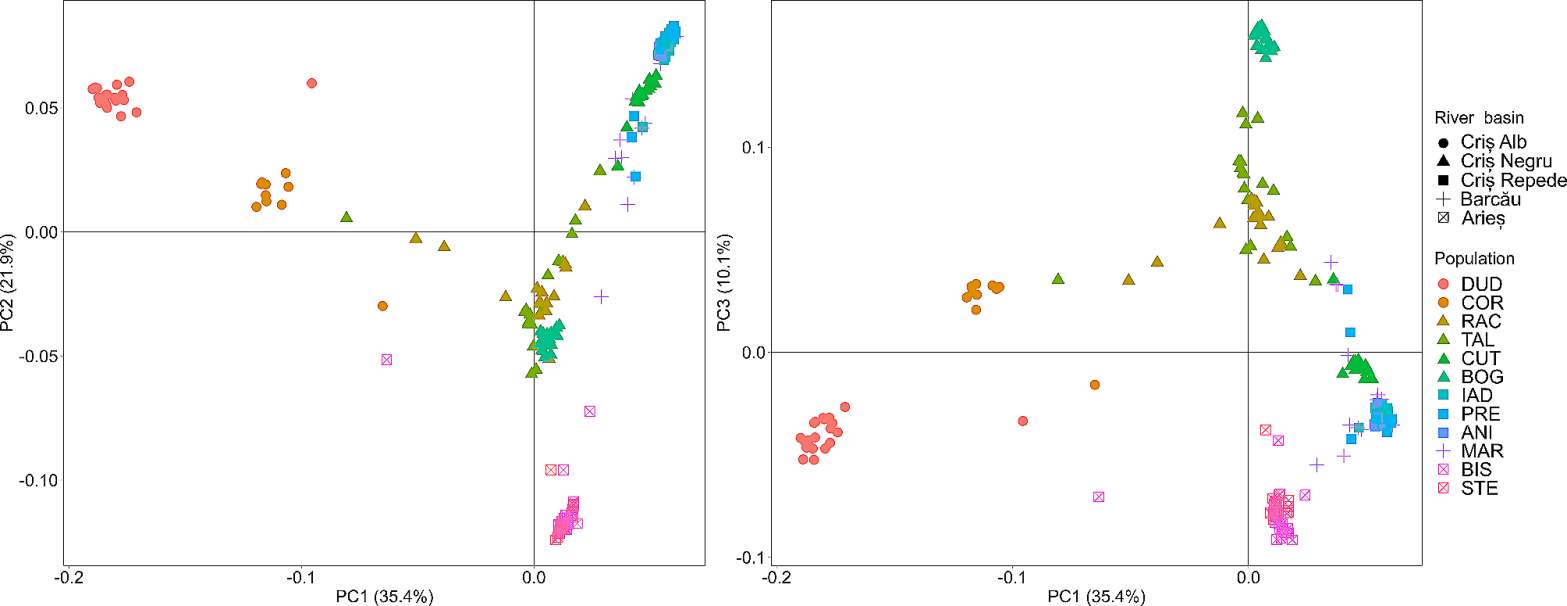
PCA. Different colours denote different populations, and different symbols the river basins to which populations belong. A – PC1 and PC2, B – PC1 and PC3. Population acronyms: DUD – Dudușoaia, COR – Corbului, TAL – Tâlniciorii, BOG – Boga, RAC – Racu, CUT – Cuților, IAD – Iadei, PRE – Preluca, ANI – Anișelului, MAR – Mare, STE – Starpă, BIS – Bistrii

The PCA analysis showed congruent results to structure analysis with K = 8, with the first two components explaining 57.3% of the variance (Fig. 4A), while PC3 explains 10.1% of the variance (Fig. 4B). Based on the variation represented in PC1, populations belonging to the river basin Criș Alb grouped separately from the rest of the analysed populations. Based on the variation from PC2, the populations of the Criș Repede river basins grouped separately, while BOG (Criș Negru basin) separated based on the variation from PC3.

**Fig. 5:**
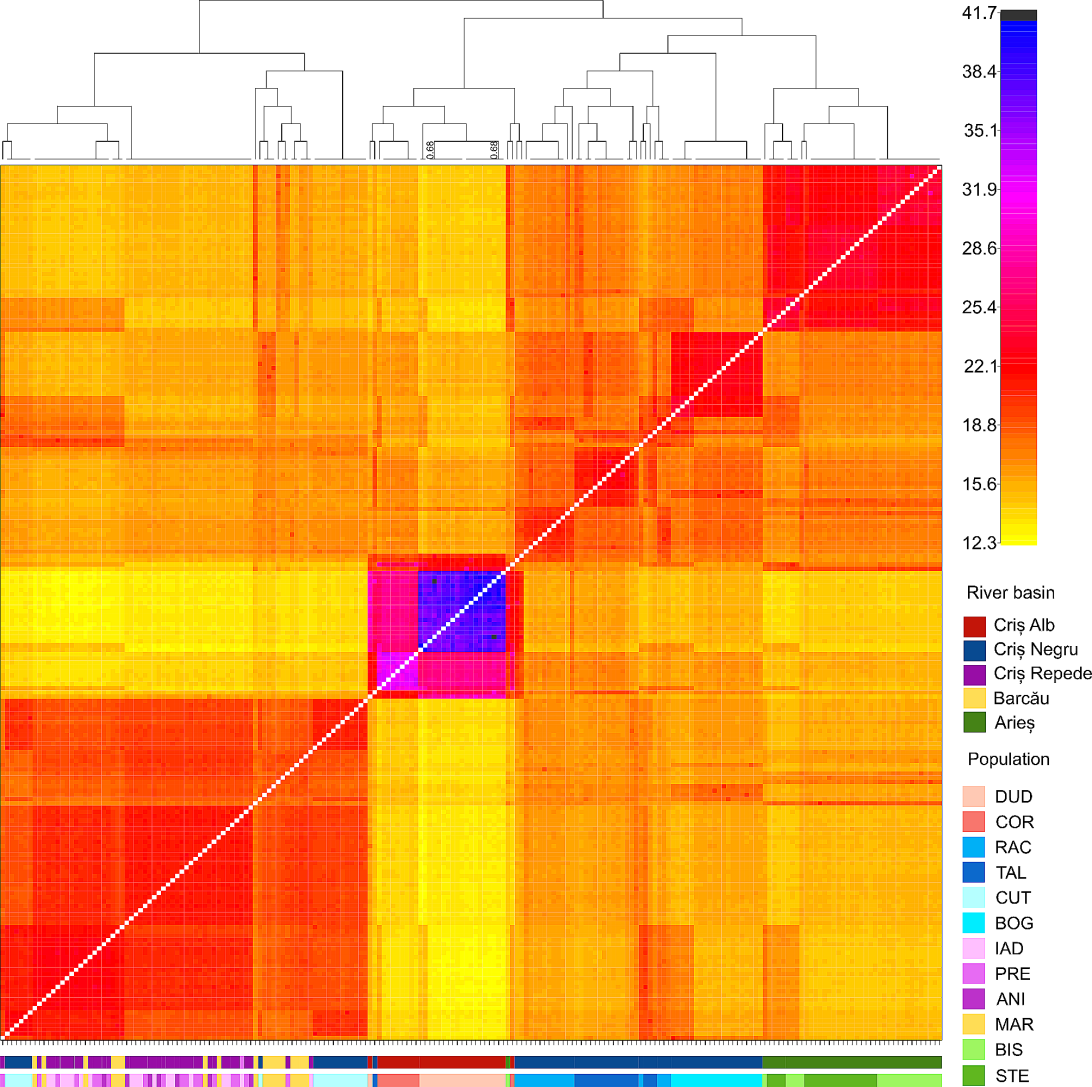
Co-ancestry matrix of pairwise genetic similarity between the individuals. Darker (blue and black) colours represent high level of genetic similarity and co-ancestry (relatedness), and light colours lower level of co-ancestry. The clustering of individuals is shown in a dendrogram on top of the matrix. Posterior probabilities values are 1 unless indicated on branches. Colour bars indicate river basins and populations. Population acronyms: DUD – Dudușoaia, COR – Corbului, TAL – Tâlniciorii, BOG – Boga, RAC – Racu, CUT – Cuților, IAD – Iadei, PRE – Preluca, ANI – Anișelului, MAR – Mare, STE - Starpă, BIS – Bistrii

The results of genetic structure based on shared co-ancestry matrices are represented in Fig. 5. Generally, individuals shared higher genetic similarity and co-ancestry with individuals from the same river basin. Furthermore, the clustering dendrogram showed clustering of the populations based mainly on the river basin. The populations from the river basin Criș Alb had the highest levels of ancestry within the same river basin compared to other populations. CUT (Criș Negru basin), MAR (Barcău basin), IAD and ANI (Criș Repede basin) populations formed one cluster and shared a more recent ancestry than with other populations. The individuals belonging to the TAL, RAC and BOG populations (all located in Criș Negru basin) grouped together. Population BOG had higher similarity within the population than with other populations. The TAL and RAC populations did not separate clearly but showed similarity between the two populations.

## Discussion

In this study, we applied reduced representation genome sequencing of 235 crayfish individuals to assess the population genomic structure and variation among the populations of the freshwater crayfish *A. bihariensis*, an endemic species to the Apuseni Mountains in Romania with the most restricted range of all freshwater crayfish species in Europe. We show that the populations’ genomic structure reflects the distribution of the populations in the river basins. Moreover, the identified low genetic diversity presents a risk for the populations and highlights the need for targeted conservation actions.

### Population genetic diversity and structure

Based on 4 875 SNPs from 12 populations covering the entire distribution range of the species, we observed an overall low genetic diversity, with almost no private alleles within populations, low heterozygosity, and low polymorphism. We confirmed our hypothesis that population structuring mainly depends on river basins. Low genetic diversity is usually attributed to small population sizes, genetic inbreeding and/or genetic drift, and bottlenecks [43]. In small and isolated populations, there is a higher likelihood of loss of rare alleles and of low migration rates [44]. This causes small effective population sizes (N_e_) and deficiency of heterozygotes, hence resulting in decreased observed heterozygosity and lower genetic variation [45]. Private alleles were found only in the DUD population, and on the river basin level, in Criș Alb and Criș Negru river basins, possibly because of the geographical isolation of these populations. Private alleles are shared among populations within river basins, but are unique among the river basins. Therefore the estimated number of private alleles is higher for the Criș Alb river basin compared to the DUD population alone. Our results showed small N_e_ for all populations, below the recommended 100/1000 rule for avoiding inbreeding and maintaining evolutionary potential [46]. These low values indicate a higher extinction risk in the long-term, especially for species with low reproductive rates [47]. Higher values of N_e_ based on heterozygote excess methods were estimated for DUD, IAD and PRE populations. However, the upper limit of the confidence interval, as well as Ne values for COR, ANI and BOG populations, show indefinite values, indicating the method cannot provide a precise estimation, possibly because of small sample size or missing information. In our analysis, F_IS_ values were around 0, which suggests random-mating in the population [48]. Furthermore, there have been no reports of mortality or severe natural events, such as drought, floods, or disease outbreaks, which could indicate a bottleneck event. Therefore, we interpret the low values of genetic diversity as the result of a combination of genetic forces, low dispersal capability, as well as habitat fragmentation.

The previous study based on five microsatellite loci, observed heterozygosity (H_O_) levels ranged from 0.325 to 0.834 in *A. bihariensis* [12]. Lower values of heterozygosity observed in our study (0.164–0.311) are expected since SNPs have lower mutation rates than microsatellites and can present only two allelic states in diploid species [28, 49]. Reports on the population genetics of other European freshwater crayfish species have mostly been based on microsatellite markers [50–53], while genome-wide studies using SNPs are still lacking. However, studies using SNPs on other species from the order Decapoda showed similar or lower heterozygosity levels compared to this study. In the lobster species *Panulirus homarus* and *Panulirus ornatus*, H_O_ ranged from 0.05 to 0.15, and the average H_O_ for the crayfish species *Procambarus clarkii* was 0.0047 [54–56]. In our study, low genetic diversity was also seen in the small percentage of polymorphic loci and private alleles in the populations. All summary statistics (heterozygosity, number of private alleles, and percentage of polymorphic loci) suggested limited genetic variation within the analysed populations. Several studies showed that low genetic diversity can lead to faster extinction [57–59]. In those cases, the population can only persist if it is able to adapt to environmental change, or able to migrate to other areas. Thus, a lower population’s ability to adapt to changing environments can increase vulnerability to environmental pressures [25]. Based on the fixation coefficient (F_ST_), which is a measure of population differentiation, the highest differentiation was observed between the populations of the river basin Criș Alb and the rest of the populations, with values above the significant level of differentiation (i.e., F_ST_ = 0.15; [60]). We detected the strongest differentiation based on F_ST_ values between the Criș Alb river basin populations and the rest of the populations. Similar results were obtained based on PCA with Criș Alb and Arieș river basin populations, both being most differentiated from the rest of the populations. fastStructure analysis revealed a most likely grouping of populations into eight genetic clusters, with single populations belonging to unique clusters. The Criș Alb river basin populations and the Arieș river basin populations form a separate cluster, indicating longer isolation periods from the other populations. Strong genetic structure on a narrow geographic range has been observed in other crayfish populations of European *Astacus astacus* [50, 61] and Australian *Euastacus bispinosus* [62] and *Euastacus armatus* [63]. This observation is expected for species with low vagility and limited dispersal capacity, such as crayfish [64].

Based on co-ancestry analysis, which indicates the degree of sharing haplotypes between individuals/populations [65], the populations group mostly according to river basins. The populations of the Criș Repede river basin and the population MAR and CUT show possible presence of gene flow. The same populations also belong to a joint genetic cluster based on our analyses, and have lower F_ST_ values indicating recent gene flow between these populations, which reduces genetic differentiation. Considering their geographic location in a karstic area, the underground connectivity between CUT population and Criș Repede river basin is highly plausible [66, 67]. Given the proximity (ca. 50 m) of the stream heads of the population MAR (Barcău river basin) to several tributaries of the Criș Repede river basin, plus proximity to the settlement Făgetu (Sălaj County), the MAR population, unique in the Barcău river basin, is likely a result of human-mediated translocation, as it has been already hypothesised based on microsatellites [12]. In the latter case, no recent or past karstic substrate could allow underground connections of the MAR population to the Criș Repede populations. Microsatellites were often used more frequently in genetic population studies. However, large SNP datasets have higher resolution power and can detect the population’s genetic structure more reliably [49]. Microsatellite markers can also overestimate the genetic variability because of the intrinsic high mutation rates, which leads to homoplasy, making it difficult to discern ancestry from independent mutations [28]. In our case, the SNPs provided a higher resolution of the population structure and showed more precision in clustering analyses than microsatellite data for the same populations, where the individuals were grouped into only one cluster [12].

### Conservation

Freshwater crayfish are considered keystone species with important ecological functions in maintaining the structure and functioning of their ecosystems [3]. Thus, the low genetic diversity of the Idle Crayfish and the small effective population sizes are particularly concerning. Reduced genetic diversity in a population can lead to decreased adaptability and decreased resilience to environmental changes, making populations less capable of effectively performing their ecological functions [68]. The decline of Idle Crayfish populations can cause cascading effects through stream ecosystems, with potentially dramatic consequences on their biodiversity [69]. Therefore, whenever present, native crayfish should be regarded as umbrella species of conservation focus in protected areas such as the Apuseni mountains, which is regarded as a biodiversity hotspot [70].

Even though *A. bihariensis* is found in multiple protected areas, there are no species-specific conservation programs in place yet, making it vulnerable especially to anthropogenic influence. The results presented here can provide important information to build an appropriate conservation program. Based on the genetic diversity of the populations, considerations of the adaptive potential of the populations are needed to make efficient conservation decisions, as populations with different genetic diversities have different capabilities of responding and minimising the effect of changing environments [71]. Identifying the genetic characteristics of the individuals and populations can inform translocations for endangered species with the goal of restoring a population and increasing its genetic diversity [72]. Considering the population structure revealed in this study, and the identified population differentiation according to their river basins, our results suggest that translocations of this species would be possible among populations within the same gene pool. Such actions could be applied between the populations in the river basins Criș Repede and Barcău, as well as within the populations of the river basin Arieș, considering their genetic similarity. However, translocation actions should also take into consideration the genetically unique populations within the river basins, BOG (Criș Negru) and DUD and COR (Criș Alb), which contribute to the overall intraspecific diversity of this species.

Reintroduction and translocation actions have been proven successful for the crayfish species *Astacus astacus* (Linnaeus, 1758) and *Austropotamobius pallipes* (Lereboullet, 1858) (*sensu lato*) whose populations were extinct due to the crayfish plague disease [73]. Although useful, the translocations can carry risks of introducing diseases, such as the crayfish plague. Since known carriers of *A. astaci* have already been identified in the Romanian’s freshwaters [19], conservation actions need to consider the potential threat of the disease introduction in unaffected populations [74]. Knowing the risks and problems that translocation carries, reintroduction and translocation actions should remain a last resort in the face of the eventual extinction of some populations. Until then, appropriate measures to preserve habitats and thoroughly prevent colonisation by invasive species should remain the main efforts in the short and medium term.

The highest conservation priority should be addressed towards the populations carrying unique genetic composition (Criș Alb and Criș Negru populations). Prospectively, as some populations might be more adaptable to potential environmental changes, further studies are needed to identify SNPs associated with more resilient phenotypes. Reference genomes can be highly useful to identify SNPs involved in specific traits or disease resistance [29, 75]. However, it is still challenging to generate high-quality reference genomes, especially for non-model invertebrate species with large and repetitive genomes, characteristic of decapods [76]. Even in species where reference genomes are available, whole genome re-sequencing of a large number of individuals needed for population genomic studies is a financially exhausting and time-consuming approach. ddRADseq is useful for obtaining genomic SNPs without a reference genome, and is appropriate for monitoring as a reproducible and low-priced method.

## Conclusion

In this study, the ddRAD approach allowed the identification of genetically distinct populations which require monitoring and priority in the conservation management of the species. Using genomic approaches for monitoring the genetic diversity of a species allows more comprehensive information than traditional single markers. Our work emphasizes the urgent need to implement a habitat preservation policy for these highly threatened populations. Moreover, we point out the need for a reference genome for this endemic species, to identify the genotypes associated with the most resilient phenotypes. In the case of *A. bihariensis*, there is an urgent need to bring this species to the forefront as a priority sequencing target to ensure the monitoring and conservation of the species.

### Electronic supplementary material

Below is the link to the electronic supplementary material.

Supplementary Material 1

Supplementary Material 2

## Data Availability

The datasets generated and analysed during the current study are available in the NCBI SRA repository, BioProjectID PRJNA1037348, sample accession numbers SAMN38171504 - SAMN38171738.
